# Accrediting private providers with National Health Insurance to better serve low-income populations in Kenya and Ghana: a qualitative study

**DOI:** 10.1186/s12939-018-0893-y

**Published:** 2018-12-05

**Authors:** Lauren Suchman

**Affiliations:** Institute for Global Health Sciences, University of California San Francisco, San Francisco, California USA

**Keywords:** Social health insurance, Healthcare access, Private providers, Low-income, Kenya, Ghana

## Abstract

**Background:**

Small private providers in low- and middle-income countries (LMICs) are well positioned to fill gaps in services to low-income populations using Social Health Insurance (SHI) schemes. However, we know little about the practical challenges both private providers and patients face in the context of SHI that may ultimately limit access to quality services for low-income populations. In this paper, we pull together data collected from private providers, patients, and SHI officials in Kenya and Ghana to answer the question: does participation in an SHI scheme affect private providers’ ability to serve poorer patient populations with quality health services?

**Methods:**

In-depth interviews were held with 204 providers over three rounds of data collection (2013, 2015, 2017) in Kenya and Ghana. We also conducted client exit interviews in 2013 and 2017 for a total of 106 patient interviews. Ten focus group discussions (FGDs) were conducted in Kenya and Ghana respectively in 2013 for a total of 171 FGD participants. A total of 13 in-depth interviews also were conducted with officials from the Ghana National Health Insurance Agency (NHIA) and the Kenya National Hospital Insurance Fund (NHIF) across four rounds of data collection (2013, 2014, 2016, 2017). Provider interviews covered reasons for (non) enrollment in the health insurance system, experiences with the accreditation process, and benefits and challenges with the system. Client exit interviews covered provider choice, clinic experience, and SHI experience. FGDs covered the local healthcare landscape. Interviews with SHI officials covered officials’ experiences working with private providers, and the opportunities and challenges they faced both accrediting providers and enrolling members. Transcripts were coded in Atlas.ti using an open coding approach and analyzed thematically.

**Results:**

Private providers and patients agreed that SHI schemes are beneficial for reducing out-of-pocket costs to patients and many providers felt they had to become SHI-accredited in order to keep their facilities open. The SHI officials in both countries corroborated these sentiments. However, due to misunderstanding of the system providers tended to charge clients for services they felt were above and beyond reimbursable expenses. Services were sometimes limited as well. Significant delays in SHI reimbursement in Ghana exacerbated these problems and compromised providers’ abilities to cover basic expenses without charging patients. While patients recognized the potential benefits of SHI coverage and many sought it out, a number of patients reported allowing their enrollment to lapse for cost reasons or because they felt the coverage was useless when they were still asked to pay for services out-of-pocket at the health facility.

**Conclusions:**

Our data point to several major barriers to SHI access and effectiveness for low-income populations in Ghana and in Kenya, in addition to opportunities to better engage private providers to serve these populations. We recommend using fee-for-service payments based on Diagnosis Related Group rather than a capitation payment system, as well as building more monitoring and accountability mechanisms into the SHI systems in order to reduce requests for informal out-of-pocket payments from patients while also ensuring quality of care. However, particularly in Ghana, these reforms should be accompanied by financial reform within the SHI system so that small private providers can be adequately funded through government financing.

## Background

One of the goals of Social Health Insurance (SHI) is to achieve Universal Health Coverage (UHC) in which a country’s entire population has access to a full range of key health services at an affordable cost [1]. At its very core, UHC requires attention to equity in health; in order to achieve this goal, countries must draw their entire population, including those most difficult to reach, into healthcare. While more developed countries with established social health insurance schemes in Europe and Asia have made significant progress toward UHC [2–4], low- and middle-income countries (LMICs) have struggled with the equity aspect of social health insurance; these schemes tend to best reach and serve the wealthy. Since private health service providers make up a significant portion of the healthcare landscape in these countries and often fill gaps in services for low-income populations [5], increasing access to these providers through SHI has great potential to bring LMICs closer to achieving UHC. However, we know little about provider experiences with SHIs, and information on the experiences of private providers is particularly spare. Similarly, we have little data on patient experience using SHI to access private care.

This article draws on qualitative data collected among private providers and their patients in two LMICs: Ghana and Kenya. Ghana is an LMIC with an established SHI system where approximately 33% of providers are in the private sector [6]. However, the health sector is currently transitioning to a more diversified system in which private providers are expected to play an increasingly large role [7]. As of 2013, 24.2% of Ghana’s population is estimated to be living in poverty [8] and a significant percentage (about 40%) of the population was covered by SHI as of 2014 [6]. In Kenya, an LMIC with an SHI scheme that has only recently become accessible to the broader population, private providers account for a significant proportion of the landscape, larger than the number of faith-based providers and NGO-backed providers combined [9]. About 36% of Kenya’s population is estimated to live in poverty [10] and less than 20% of the population is covered by health insurance, although over 88% of those covered are enrolled in the SHI system [11]. In this paper, we pull together data collected from private providers, patients, and SHI officials in both countries to answer the question: does participation in an SHI scheme affect private providers’ ability to serve poorer patient populations with quality health services?

### Accrediting providers for quality

As defined by the International Society for Quality in Healthcare, “accreditation” is: “A public recognition by a healthcare accreditation body of the achievement of accreditation standards by a healthcare organization, demonstrated through an independent external peer assessment of that organization’s level of performance in relation to the standards” [12]. Accreditation consists of four basic components: development of an accrediting organization; development of accreditation standards and the criteria to meet these standards; implementation of a survey process; and incentives and disincentives [12].

While developed countries such as the United States have long histories of hospital accreditation, low- and middle-income countries (LMICs) have only started accrediting healthcare providers more recently as part of efforts to achieve UHC [13]. Accreditation tied to pre-determined standards of quality is therefore particularly attractive to countries that aim to achieve UHC through SHI while improving their overall health system, allowing government insurers to “purchase for quality” [12]. However, while accreditation does seem to have an effect on quality, these effects are uneven across different areas of care and it is unclear if improvements made to meet accreditation standards ultimately result in improved health outcomes [14–17].

Although there is little literature pertaining specifically to the accreditation of private providers, Slack and Savedoff [18] suggest that governments purchasing health services from private providers in LMICs should determine ahead of time how much quality needs to be monitored depending on each private facility’s level of independence; with increasing levels of autonomy, private facilities should be more closely monitored. Indeed, some research conducted in Ghana found that the majority of those who became accredited by the NHIA between 2009 and 2012 achieved scores that were barely passing, indicating a relatively low level of quality. Among all types of private providers (e.g. pharmacies, maternity homes), clinics had the highest number of failed applications for accreditation [19]. However, this study did not give any indication of whether or not these scores were a relevant indicator of quality as compared to public health facilities. We were not able to find any literature on accrediting private providers in Kenya.

### National Health Insurance Accreditation in Kenya and Ghana

Ghana’s National Health Insurance Scheme (NHIS) went into effect in 2003, making it one of sub-Saharan Africa’s oldest social health insurance schemes. The NHIS is meant to cover all Ghanaians with the same package of services [20, 21]. This package includes both inpatient and outpatient services, including treatment for malaria, certain medications, oral health, and eye health. It also includes emergency care and maternity services, although it excludes some specialty services such as cancer treatments, HIV treatment, and dialysis (www.nhis.gov.gh). Most enrollees pay premiums to participate in the scheme, although populations such as the poor and the elderly are exempt from payment [22].

According to the NHIS website (www.nhis.gov.gh), in order to become accredited private providers must first be registered with the state and be in good standing. All providers (public, private, and faith-based, including pharmacies) must: have been in operation for at least 6 months; have adequate facilities and human resources for service provision; accept the NHIA’s quality assurance standards; and have a quality assurance program in place. These facilities then complete an application and submit it to the NHIA with a fee, after which an NHIA representative visits the facility for an in-person assessment and makes their recommendation whether or not to accredit the facility. This recommendation includes a grade of the facility’s overall performance, and this grade is published online so that clients have access to information about differences in quality at accredited facilities.

During the accreditation process, providers also are classified by type and level, which determines the reimbursement rates they can receive from the NHIS as well as the services and drugs for which they can claim reimbursement [23, 24]. Under this system, smaller facilities receive lower reimbursement rates and are able to claim reimbursement for fewer services than larger hospitals. This is based on the assumption that larger facilities have higher operational costs, but is also meant as a quality check; presumably, smaller facilities are less likely to offer services they aren’t qualified or equipped to provide if they cannot be paid for these services by the NHIS. Providers are then paid on a fee-for-service basis in which they submit claims for services rendered and receive reimbursement from the government, ideally within 1 month [22]. However, a growing body of evidence points to significant delays in payment [25, 26]. Despite these delays, the NHIA estimated that approximately 40% of accredited providers were from the private sector as of 2013 [27].

Although Kenya’s National Hospital Insurance Fund (NHIF) has been in existence since the 1960s, it has largely served the country’s civil service and only recently became available to workers in other sectors, both formal and informal [28, 29]. According to the NHIF website (www.nhif.or.ke), benefits include a range of both inpatient and outpatient services, such as general consultations, diagnostics, and medications. NHIF also covers some specialty services for chronic conditions, such as renal dialysis. Enrollees pay monthly premiums to maintain membership in NHIF, which comes to about 500 Kenyan Schillings per month for those in the informal sector.

The NHIF website also details the process for providers to become accredited. Providers must first be licensed with the Kenya Medical Practitioners and Dentists Board and then undergo an on-site assessment. This comprehensive assessment covers such aspects of the facility as infrastructure, human resource management, availability of equipment, and infection prevention measures. Accreditation contracts are then given according to provider type. At Category A facilities (government hospitals), patients can access all services for free, provided they have maintained their enrollment in NHIF. Private and mission hospitals fall under Category B, in which patients are covered for most services, but may be required to pay for specialty services, such as surgery. Only private providers quality for Category C contracts in which the NHIF covers fewer services than those included in Category A and B. Unlike in Ghana, Kenyan providers accredited by the NHIF are reimbursed on a fee-for-service model only for inpatient services, while outpatient services operate on a capitation model. Under capitation, providers are paid a monthly sum based on the number of patients registered to their particular clinic. This payment is meant to act as a financial risk pool within an individual facility, assuming that some patients will never show up for care in a given month, while others may require more expensive services [30].

### Covering poor populations with National Health Insurance

Numerous studies in both Ghana and in Kenya show that low-income populations are far less likely to benefit from SHI than their wealthier counterparts. In Ghana, while the NHIS has helped to reduce “catastrophic” health expenditures, which disproportionately affect poor populations, studies have found that the overall distribution of NHIS benefits tends to favor the rich [22, 31–34] . Similarly, evidence from Kenya indicates that populations that tend to be lower income, such as women [35] and those employed in the informal sector [36, 37] are unlikely to enroll in the NHIF. Since outpatient services have only recently become available to other sectors beyond the civil service [38], this more recent access to health insurance among low-income populations may explain studies that show limited understanding of how insurance works both among insured and uninsured populations in Kenya [39]. This problem of lack of information and understanding related to NHIF coverage may be exacerbated by variable and inconsistent communication regarding these schemes from the government [11].

As Agyepong et al. [40] point out, differential access to SHI is a result of the confluence of a number of factors related to economic, political, and sociocultural context. In addition to lack of information, as noted in Kenya above, one of the most obvious and most common barriers to SHI access for poor populations is cost. A number of studies have found that enrollment fees prohibit potential beneficiaries both from registering with the Ghana NHIS in the first place and also from re-enrolling annually [41–43] . This is coupled with perceptions that services covered by the NHIS are low quality and therefore not worth the cost or difficulty of enrollment to begin with [33, 44, 45]. Similarly, inability to pay insurance premiums has been cited as a barrier to NHIF access in Kenya [11, 39], where out-of-pocket payments were a significant percentage of health expenditure from 2002 to 2008 [46].

In addition to formal premiums, some research has found that patients in Ghana are asked to pay out of pocket for services that should be covered by NHIS. Since the NHIS has a history of significant delays in reimbursing providers for services, providers justify requests for out-of-pocket payment by pointing out that they would not otherwise have enough money to run their clinics [25]. In other cases, providers charged for NHIS-covered services because they felt the reimbursement rates were inadequate and did not fully cover the cost of the service [47]. Although we did not find similar evidence in the literature on Kenya, we suspect this is due to the relatively recent extension of NHIF services to those working outside the civil service; a provision that has greatly expanded NHIF access to the general population. Certainly, though, incidents of bribery are well documented in the Kenyan medical system [28].

In addition to direct costs incurred either at the point of enrollment or in the clinic, indirect costs also are a barrier to NHIS use among poor populations. Indeed, one of the most common indirect costs cited in the literature is the cost of transportation; the poorest individuals in Ghana tend to live the farthest from health facilities compared to their wealthier counterparts [48] and distance from a health facility was found to be a common barrier to use, despite NHIS enrollment status [49]. Further, as Agyepong et al. [40] point out, the combination of direct and indirect costs related to using NHIS coverage can add up for a patient who needs to pay an annual enrollment fee, pay again for transportation to the clinic, and again when asked for out-of-pocket payments for services. Agyepong et al. suggest that these compounding fees undermine the government’s branding of the NHIS as a “free” service, ultimately making the service less accessible in addition to eroding trust in the system among lower-income beneficiaries.

While there is evidence from Kenya that, within the private sector, private not-for-profit clinics have a specifically pro-poor bent [50] and some have recommended that the government better regulate and resource private clinics in resource-poor urban settings [51], there is a dearth of literature on provider experiences with SHI systems, and particularly the experiences of the private providers who make up a significant proportion of the healthcare landscape in both Kenya and in Ghana [52]. A 2011 study conducted in Ghana [53] found that patients paid more at private facilities, but felt these providers were more trustworthy and offered better care. Similarly, a household survey conducted in Kenya found respondents believed that public health facilities had poor quality of service [39]. However, the poorest of the insured population in Ghana is most likely to access care at the public Community-based Health Planning and Services (CHPS) compounds, as opposed to private clinics [54]. Regardless of provider type, respondents in Ghana believed that patients paying out of pocket (without insurance) received better treatment than those paying with NHIS [53], and evidence from Kenya similarly suggests that providers may discriminate against NHIF beneficiaries as compared to patients paying out of pocket [11].

In sum, SHI systems in both Kenya and Ghana need to do a better job of reaching low-income populations if they hope to achieve more equitable healthcare coverage in their respective countries. Since private providers are more numerous and more widely dispersed than their public counterparts in both Kenya and Ghana, there is great potential for private providers to assist in filling this equity gap. However, while accrediting private providers under SHI schemes should make services more financially accessible while also ensuring a minimum level of quality across a disparate group of providers, we have little information on private provider experiences with accreditation and how these experiences affect both cost and quality of care. In this paper, we explore private provider experiences with accreditation and balance this data with patient perspectives regarding accessibility, cost, and quality of services under NHI schemes, as well as the perspectives of NHI officials themselves. By bringing this data together, we aim to determine the extent to which participation in an NHI scheme affects private providers’ ability to serve a wider patient population with quality health services in Kenya and Ghana.

## Methods

The data for this paper were collected as part of the qualitative evaluation of the African Health Markets for Equity (AHME) program in Ghana and Kenya. AHME aims to link supply (private providers) with demand (clients) in order to shift health markets toward providing quality healthcare to low-income patients in Kenya and Ghana. With this goal in mind, AHME works through social franchises, networks of providers that apply the principles of commercial franchising to health services [55], to provide a package of quality improvement and financing interventions. The AHME intervention package includes: social franchising; SafeCare, a step-wise quality improvement program managed by the PharmAccess Foundation; the Medical Credit Fund, a business training and loans program also managed by the PharmAccess Foundation; and NHI accreditation assistance. On the client (demand) side, AHME also provides support for activities to identify and enroll low-income populations into the NHIs.

The qualitative dataset analyzed below consists of in-depth interviews with private sector healthcare providers at facilities that were members of one of the AHME partner social franchises, as well as facilities that had been approached to join the franchise network but declined. In addition, we conducted exit interviews with clients at both the franchised and non-franchised facilities, as well as focus group discussions with community members in AHME catchment areas. We also conducted interviews with select officials who worked with AHME at the National Health Insurance Authority (NHIA) in Ghana and the National Hospital Insurance Fund (NHIF) in Kenya. Data were collected in three rounds among providers (2013, 2015, and 2017) and NHI officials (2014, 2016, 2017), and in two rounds (2013 and 2017) among patients. Focus group discussions took place in Round One (2013) of data collection to gather background information on the local healthcare landscape and unmet needs, but were not repeated. Measures were undertaken across the three rounds of sampling, data collection, and analysis to increase the validity and reliability of the study. These measures are detailed in each of the relevant sections of the study methods.

### Sampling

During each round of data collection, the AHME social franchising partners, Marie Stopes International (MSI) and Population Services Kenya (PSK), provided the research team with lists of providers franchised under the Amua (MSI, Kenya), Tunza (PSK) and BlueStar (MSI, Ghana) networks. During Rounds Two (2015) and Three (2017) of data collection, the franchise partners also provided lists of providers who had been contacted to join the franchise, but had declined. These clinics were included in the sample to provide a point of comparison against which the research team could better determine the effects of the AHME interventions. Using these lists, we used a purposeful criterion sampling strategy [56] to design a sample that represented providers with a mix of experiences with the AHME intervention package, including non-franchised providers who had no experience with the interventions (Table 1). In order to capture potential effects of the NHI accreditation assistance intervention, in Rounds Two and Three we also selected facilities based on their NHI accreditation status. Although we aimed to equally represent both accredited and non-accredited facilities, this was particularly challenging in Ghana. NHIS accreditation is very common among all providers in Ghana, which made it difficult to sample non-accredited providers. Interviews were conducted with providers in a range of facility types across six regions in Kenya (Nairobi, Eastern, Coast, Central, Rift Valley, Kajiado) and three regions in Ghana (Greater Accra, Volta, Ashanti) during the three rounds of data collection. These regions were partially chosen opportunistically, given that the franchise networks were still expanding and had little or no representation in some regions when data collection was conducted. However, the research team aimed to reach a mix of metropolitan and rural areas with this sample, assuming that both providers and patients would have had differential exposure to both the AHME interventions and the NHIs depending on their proximity to an urban center, such as Nairobi or Mombasa in Eastern Kenya. In some cases, a region was chosen specifically because the NHI system was expanding or testing a new program in that area, such as the NHIS’s capitation pilot in Ashanti. Within each facility, we instructed field staff to request an interview with the owner of the facility or a staff member with the greatest knowledge of facility management to best capture provider experiences with the AHME interventions and with the NHIs.

**Table 1:** Provider sample

		Round 1 (2013)		Round 2 (2015)		Round 3 (2017)	
		Kenya	Ghana	Kenya	Ghana	Kenya	Ghana
Total		24	23	52	27	50	28
Franchised clinics		24	23	45	24	30	20
	Tunza (PSK)	14	*N/A*	17	*N/A*	15	*N/A*
	Amua (MSK)	10	*N/A*	19	*N/A*	15	*N/A*
	MCF	N/A	*N/A*	6	4	27	9
	SafeCare	N/A	*N/A*	15	17	28	14
		*Accredited*	*Accredited*	*Accredited*	*Accredited*	*Accredited*	*Accredited*
		N/A	N/A	18	19	19	27
Non-franchised clinics		*N/A*	*N/A*	7	3	20	8
		*Accredited*	*Accredited*	*Accredited*	*Accredited*	*Accredited*	*Accredited*
		N/A	N/A	0	1	*9*	*8*

To capture patient perspectives on clinic experience as well as experiences with the NHIs, we held two rounds of exit interviews with clients in 2013 and 2017 (Table 2). Since the AHME Qualitative Evaluation is part of a larger mixed methods evaluation that includes a Randomized Controlled Trial (RCT) in Kenya, we sought alignment with RCT sampling criteria for client exit interviews. Thus, client interviewees in both countries were screened for: gender (women only); age (between 18 and 49 years of age); and number of children (interviewees were required to have at least one child aged 5 years or less). In addition, respondents had to be exiting one of the selected franchised or non-franchised clinics. Clients also were selected for NHI enrollment status with an aim to sample NHI-enrolled and non-enrolled patients equally, although this proved difficult in Ghana where most clients screened had NHIS coverage. We did not collect data on NHIS enrollment status among clients in Ghana during Round One because at that point the role of NHIS within the AHME initiative was not yet confirmed. In order to better capture data related to AHME’s goal of reaching poor populations, in the last round of data collection (2017), we sought to over-sample patients exiting clinics located in low-income areas.

**Table 2:** Client sample

		Round 1 (2013)		Round 2 (2017)	
		Kenya	Ghana	Kenya	Ghana
Total		26	20	30	30
Franchised clinics		26	20	20	23
	Tunza (PSK)	19			*N/A*
	Amua (MSK)	7			*N/A*
		*NHI-enrolled*	*NHI-enrolled*	*NHI-enrolled*	*NHI-enrolled*
		13	N/A	14	27
Non-franchised clinics		*N/A*	*N/A*	10	7
		*NHI-enrolled*	*NHI-enrolled*	*NHI-enrolled*	*NHI-enrolled*
		N/A	N/A	4	6

In order to gather data on the local health landscape and unmet community needs, we conducted Focus Group Discussions (FGD) with community members during Round One (2013) (Table 3). In order to maximize the likelihood of capturing descriptions of market effects from the AHME interventions, we restricted selection to community members, both women and men, in the areas surrounding a few key providers who also participated in interviews. The FGDs were stratified by gender and by age group (18–24, 25–35, and 36–49) in order to form more homogenous groups and facilitate conversation. We also restricted FGDs to respondents with at least one child, as a number of questions dealt with child health scenarios. Since we determined that the 2013 FGDs did not provide enough relevant data to determine the larger market effects of AHME, we decided not to repeat FGDs in subsequent years.

**Table 3:** Community member focus group sample

	Kenya		Ghana	
Total participants	99		72	
# of groups	10		10	
Age group	Female	Male	Female	Male
18–24	2	1	1	0
25–35	2	2	3	2
36–49	2	1	3	1

Finally, in order to provide a higher-level perspective on provider and patient experiences, we interviewed officials at the NHIA in Ghana and the NHIF in Kenya (Table 4). Since the AHME project has a relationship with both organizations, we selected officials who worked directly with AHME. This selection further ensured that the officials would have some interest and experience in both working with private providers and targeting poor populations for NHI enrollment.

**Table 4:** NHI official sample

	Round 1 (2013)	Round 2 (2014)	Round 3 (2016)	Round 4 (2017)
Kenya	0	2	2	3
Ghana	0	0	4	2

### Data collection, processing, and analysis

The Qualitative Evaluation team partnered with Innovations for Poverty Action (IPA), a research organization based in New Haven, CT with country offices across the globe to collect data in both Kenya and in Ghana. IPA recruited field interviewers who were then trained by the UCSF Qualitative Evaluation team working with IPA staff. Training included an overview of qualitative interviewing, informed consent, and field ethics with close review and practice of the interview guides for each respective round of data collection.

Data collection took approximately 1 month in each country during each round. Field staff traveled to clinics where providers had already been contacted by IPA and agreed to participate in an interview. Interviewers then obtained informed consent from the providers prior to conducting interviews that lasted approximately 60 min each. During each round of data collection, providers were asked about their experiences with the AHME interventions and their knowledge of or desire to join any interventions in which they were not currently participating. In Rounds Two and Three (2015, 2017) of data collection, providers also were asked about their perceptions of and experiences with the NHIs. These questions were not included in Round One (2013) because NHI accreditation was not yet a component of the AHME program. Data related to provider experiences with the NHIs is most relevant to this article, as well as some data on experiences with the AHME interventions targeted to improve clinic quality: SafeCare and social franchising.

During client exit interview rounds (2013 and 2017), interviewers obtained permission from providers to approach clients leaving their clinics for screening. Clients who both met the screening criteria and agreed to an interview were consented and interviewed in an area of the clinic that was relatively private and quiet. In both rounds of data collection, clients were asked a series of questions related to their experience at the clinic and affordability of services, their health-seeking behaviors, and their knowledge of the local healthcare landscape. In Round Two (2017) of the exit interviews, clients with NHI coverage were specifically asked about their experience using NHI in the clinic, while patients without NHI coverage were asked to describe what they knew about the NHI scheme. These questions were not included in Round One of data collection, because the AHME partners had not yet decided to focus on the NHIs as the main mechanism to connect quality providers with poor patients. Data related to client experience with and knowledge of the NHIs, as well as clinic experience, are most relevant to this article.

To recruit potential franchise patients for the focus group discussions, the qualitative study coordinator and a study researcher contacted community leaders (assemblymen, local chief, or other relevant local leader) several days before the planned discussions to introduce the study, identify appropriate respondents, and decide on a location. Participants were asked questions related to their knowledge of and experiences with local healthcare and unmet needs in their communities. These focus groups were not repeated in later rounds of data collection, because they yielded little useful information regarding the potential for the franchised AHME clinics to better attract different types of patients. However, some of the data related to healthcare costs is relevant to this article.

Since the NHI official sample was much smaller than the other research populations and all interviews could be conducted in English, these interviews were completed by the UCSF research team. Key contacts within AHME would first introduce the officials to the research team via email and the researchers would then follow up to schedule interviews during a time when they were able to be in country to conduct the interviews in person. The NHI officials were asked questions related to the priorities of their organization, their experience working with private providers, and the opportunities and challenges they faced both accrediting providers and enrolling members.

All interviews and FGDs were recorded using digital recorders. These interviews were conducted in the language the respondent was most comfortable using, with some interviews being conducted in the local language, some in English, and some a mixture of the two. In expectation that all respondents would not be comfortable conducting a full discussion in English, field guides for providers and patients were first developed in English and then professionally translated into the local language (Swahili in Kenya and Twi in Ghana) ahead of data collection to ensure that the translations accurately captured the intended meanings of the original guide. Field staff were natives of Kenya and Ghana who were fluent in both English and the local language. During their training, these field staff were guided through translation of key terms in the field guides to further guarantee accurate translation during the interviews. Recordings were then translated and transcribed simultaneously by a team of professional transcriptionists who were also natives of Kenya and Ghana, and had been trained on key terms. IPA research assistants in Ghana and Kenya were responsible for back-checking interviews, including ensuring translation accuracy. As a result, any inaccuracies in data collection due to incorrect or incomplete translation should be negligible at most. All interviews with NHI officials were conducted in English and were transcribed by a consultant based in Kenya who had also worked on transcribing provider and patient interviews.

After the back-checking process was concluded, IPA transferred the transcripts to UCSF for analysis. The transcripts were then coded by the UCSF team with some assistance from IPA using Atlas.ti, a widely-used qualitative analysis software. We used an inductive, thematic approach to coding and analyzing the interviews because, particularly in the case of Kenya, there was little existing literature on private providers’ experiences with the NHIs from which to derive prior theories. An initial coding scheme was created based on thematic coding of a sub-set of the interviews from each country and each interview was coded using an open coding approach, in which codes were derived from the data. Common codes were identified across the interviews and grouped into code families and sub-codes. The UCSF Qualitative Evaluation team then reviewed the initial codebook together to ensure common understanding of codes and consistency in code application. Codes were refined over the course of the three rounds of data collection to allow for new priorities in analysis while ensuring continuity across rounds.

### Ethical review

We received initial approval with “Exempt” status from the Institutional Review Board of the University of California San Francisco for the AHME evaluation on 13 June 2013. We also received Ghana Health Services Ethical Review Committee (ERC) approval on 28 June 2013 and Kenya Medical Research Institute (KEMRI) approval on 28 October 2013. Prior to each round of data collection the Qualitative Evaluation team submitted amendments and received approval from all three review boards for any changes made to our protocol. Approvals for Round 3 (2017) of data collection was received on 16 November 2017 from ERC, 12 December 2017 for UCSF, and 17 January 2017 for KEMRI.

### Limitations

Since the data analyzed here was drawn from specific franchise networks, the findings from the AHME Qualitative Evaluation cannot be generalized to all franchised providers and their clients in either Kenya or Ghana. Further, the evaluation team relied on AHME partners MSI and PSK to identify and gain access to franchised providers. While this tactic was important to gain provider trust, it likely led to a degree of courtesy bias because providers associated the evaluation team with the franchises. As a result, providers may have been more likely to reflect positively on their experiences with the franchise and related interventions than they would have if they understood the interviews were being carried out by an objective third party. The same may be true for client exit interviews. Second, because all data was self-reported, we did not review provider registers, and the NHI official sample population was relatively small, the evaluation team could only verify provider and patient claims regarding challenges with the NHI schemes to a limited extent. Since public health facilities were not included in our sample, we also had no way to verify patient reports of mistreatment in the public health system. Finally, because the Qualitative Evaluation of the AHME project is divided into two parts, a process evaluation and a provider/patient evaluation, there is limited overlap between the questions we asked of NHI officials and those we asked providers and patients. We have done our best to cite relevant data from the NHI officials below.

## Results

Officials interviewed from both the NHIA and NHIF agreed that National Health Insurance is critical to the overall health of their population and enrolling poor populations into the schemes was a priority for both agencies to reach UHC. Further, accrediting private providers in particular was seen as an important aspect of achieving UHC as well. In fact, officials in both countries said local SHI representatives were encouraged to recruit private clinics in areas that were under-served by public health facilities.

*Sometimes our staff may just go appoint a person because you are a private facility within this locality, can you please apply [for NHIS accreditation]?…Maybe there is only one clinic, there is only one pharmacy shop or pharmacy in the locality ourselves can appoint them to say that we think if you can apply it will help our members.* (NHIA Official, Ghana)

In Kenya, this practice was specifically linked to reaching poor populations:

*We do have a report, and from that we were able to see that majority of [wealth] quintile one [the lowest wealth quintile] that we targeted, they normally access government facilities and their issues was just the distance. Especially outside of urban areas.* (NHIF Official, Kenya)

However, while SHI officials expressed a strong desire both to accredit more private providers and to reach more patients with SHI coverage, all were aware of the many challenges both providers and patients faced in gaining meaningful access to the benefits of National Health Insurance. These challenges were corroborated by our interviews with providers and patients, and included difficulties navigating complex accreditation processes, inadequate or delayed reimbursements, and difficulties operating under capitation schemes among providers. Patients faced logistical challenges at registration sites and complained of being charged out of pocket even when paying with their SHI card; difficulties that are particularly burdensome for low-income populations.

However, both providers and patients did receive some meaningful benefits from the SHIs as well. Providers often found that their client load increased as a result of becoming accredited and some felt they were better able to serve poor clientele. Patients felt that SHI coverage gave them greater access to a variety of providers, including the quality private providers they preferred. These perspectives are detailed below and interwoven with data from the SHI officials themselves to lend an institutional perspective to the benefits and challenges experienced on the ground.

### Accreditation/enrollment

#### Providers: Why become Accredited & Experiences with the accreditation process

Particularly by the last round of data collection (2017), providers commonly cited market pressure as a strong motivator to seek out SHI accreditation. It was common for both Kenyan and Ghanaian providers to report that they sought out SHI accreditation due to client demand, considering accreditation critical to business viability and also to serving low-income populations. While this trend was relatively consistent across rounds of data collection in Ghana, in Kenya client demand became a strong motivator only recently, following the extension of outpatient coverage to beneficiaries other than civil servants in 2015. By attracting more clients through their SHI accreditation, providers in both Kenya and Ghana most often reported that they hoped to increase their clinic revenues.

In addition to providers’ perceptions that becoming SHI-accredited would generally increase client flow, a number of providers specifically mentioned that they wanted to better serve low-income clients while also maintaining a viable business. In Round 1 of data collection some providers in Kenya expressed concern about finances, which were constrained by the low-income patient population they served. Indeed, providers in both countries cited clients’ frequent inability to pay as a reason why they decided to apply for SHI accreditation in the first place. When faced with low-income patients who couldn’t pay, providers in both countries reported negotiating prices with these patients or treating them for free; both options that can be financially challenging for small private clinics that already operate on constrained budgets. Thus, providers in both countries suggested that SHI accreditation was in fact essential to maintaining their business viability, particularly for clinics serving a low-income population. As one provider in a low-income neighborhood in Ghana said:

*If you not accepting, look…look, look at the people who are around, they are all poor…* ***Without NHIS you can’t operate in a clinic they better shut down your clinic, yeah****.* (Doctor at a BlueStar clinic, Greater Accra, Ghana)

This provider maintained that, because her clinic was operating in a low-income neighborhood where people had little money to pay for healthcare, financing from the NHIS allowed her to keep her clinic open. One NHIA official echoed this provider’s statement, saying:

*It’s demand driven…So, you apply when you think you need NHIA to support in terms of getting people coming to your facility…So, depending on where the [facility] is located, and let me say that in this country…you realize that most private facilities have difficulty getting members. People coming pay out of pocket because their fees are a bit on the higher side. And so most of them are rushing [to become accredited] because they noticed that without NHIA they can’t survive.* ***Especially depending on where the facility is located if the poverty level is very high they don’t have NHIA, you are not a member of NHIA, you are not accredited or credentialed it’s likely that your facility may collapse****.* (NHIA Official, Ghana)

Further, among providers who hadn’t previously served low-income populations, some felt that SHI accreditation enabled them to do this. As one provider in Kenya said:

*Coz people used to see us initially like this place is expensive it is for the rich. But due to NHIF now we encounter all groups. All classes. So, if somebody goes there he or she says but I was treated at [clinic name] And they are like, with which card, NHIF. So the… the… that thing of a hospital for the rich…It’s now over.* (Auxiliary Nurse at a non-franchised clinic, Nyanza, Kenya)

In this case, the provider felt that her clinic’s reputation had changed as a result of accepting NHIF. While this clinic had formerly served a wealthier population, she claimed that their clientele had changed after becoming NHIF accredited, and they were able to serve lower-income clients paying with an NHIF card.

Across all three rounds of data collection, most providers in Ghana had already been accredited with the NHIS for a significant period of time. These providers generally cited few challenges with the accreditation and renewal processes, noting that NHIS officials would even visit a clinic well in advance when their accreditation contract was due to expire to give them sufficient time to renew. Some newly accredited providers cited long wait times of up to 2 years to receive their accreditation, but generally clinics were able to renew their contracts within 3-to-6 months.

In contrast to the providers in Ghana, most Kenyan providers did not have NHIF accreditation in the early rounds of data collection and became more interested in pursuing accreditation more recently. However, Kenyan providers often lacked information on the accreditation process. This lack of information discouraged those providers who found the process daunting. Among those who had attempted to go through the accreditation process, Kenyan providers regularly cited difficulties with hold-ups in the accreditation process and having to “push” to keep the process moving forward.

#### Patients: Why Enroll & Experiences with enrolling in/renewing SHI membership

Like the providers interviewed, patients were generally positive when discussing the potential benefits of Social Health Insurance and their comments indicated that the SHIs were fulfilling their primary purpose of providing financial protection for enrollees. Clients in both Ghana and Kenya were aware that SHI coverage is useful for reducing costs and making healthcare more affordable, and commonly cited this as a benefit and a reason for enrolling. In particular, patients commonly suggested that this increase in affordability translates to an increase in healthcare accessibility; this suggestion was sometimes linked specifically to the benefit of affordable maternity services. Indeed, clients said that they were more likely to visit the clinic when they weren’t feeling well or had an antenatal appointment because they knew NHIS would cover their costs:

*Because with the card, if I go anywhere and I’m registered – when I’m sick, I just take it along with me.* ***With that you don’t fail to go to the hospital because of lack of money, right?*** (Patient at a Bluestar clinic, Ashanti, Ghana)

*Because if you have it and sickness strikes, even if you do not have money you will not worry because the card will pay. You’ll just be sorted.* (Patient at a non-franchised clinic, Coast, Kenya)

Further, a number of patients in both countries saw a link between the SHIs and access to healthcare for people with low income. Patients in Kenya often noted that NHIF is helpful in cases where someone has “no money” or a “lack” of money and one patient noted that NHIF was especially useful for the “under privileged people.”

While fewer Kenyan patients were enrolled in NHIF than their counterparts in Ghana, those who were enrolled cited few challenges in the enrollment process other than cost.

*Interviewer: Okay. And what is so challenging about applying for NHIF? What is difficult?*

*Respondent: Money. [Laughs] It is only money.* (Patient at a Tunza clinic, Central, Kenya)

However, this may be a result of the automatic enrollment many Kenyans receive through their work. In addition, a number of women interviewed in Kenya reported that their husband was the primary NHIF cardholder for their household, indicating they may not have gone through the enrollment process themselves.

Once enrolled, some clients again cited cost as a barrier to maintaining their NHIF membership. Patients reported allowing their enrollment to lapse because they could not make the monthly payments. In these cases, the NHIF requires patients to pay a penalty for a lapse of less than 1 year, while those who have neglected to pay for more than a year must renew their membership and wait 60 days for re-activation. These requirements made it even more challenging for clients to maintain or renew enrollment during times of financial hardship. A few Kenyan patients also said they didn’t see enough financial benefit in continuing to pay for insurance when they still incur charges in the clinic, though it was not clear if they were referring to legitimate charges or to informal charges levied by providers on top of their NHIF payment.

In contrast to the cost barriers experienced by patients in Kenya, Ghana interviewees often cited logistical challenges as a barrier to renewing their NHIS membership. The most commonly cited cause for allowing enrollment to lapse was long wait times at NHIS registration centers. Clients often reported leaving their home at 3:00 or 4:00 am to join the line for registration and then waiting a full day to go through the process. Some spent all day waiting only to be told to return the next day when the machines used for registration were experiencing connectivity issues.

*[The NHIS officers] asked us to wait. So, we waited for a while, and the computer was going off and coming back. So, they told us they were wasting our time, so we should go, but when the computer begins to work well they will work on everything.* (Patient at a non-franchised clinic, Greater Accra, Ghana)

One NHIA official confirmed that connectivity has been a problem, particularly for mobile registration sites.

*You know, it’s a developing country and that in most of the remote areas you will not find connectivity. And that also affected somehow when we were doing the enumeration [to identify and enroll poor populations into NHIS]…In some instances they would do the same …They will need to travel or walk to a distance where they can get connectivity to get the system a bit [inaudible] before they can go back and continue.* (NHIS Official, Ghana)

In addition to the connectivity challenges, a couple of clients reported having been asked for bribes to move the process along more quickly and they were clearly critical of this practice. Conversely, several women noted that they were pushed to the front of the line and enrolled for free when they were pregnant; a result of the NHIS Free Maternity Services.

### Benefits of the SHIs

#### Providers: Perceived benefits of accreditation

As providers anticipated, there was general consensus that SHI accreditation did increase client volume. In addition, some providers reported attracting more patients who would not have been able to pay for services otherwise:

*Because when we started in the initial stages and I think the first year, we were solely cash and carry and the attendance was not much but as we had NHIS accreditation, the attendance went up…We were expecting an increase in NHIS patients, those who cannot afford to pay cash and carry, yes. That is why they have the NHIS cards, and those are the people we were targeting, the people in the communities around and the villages around. That majority of them are having NHIS cards and those were the people we were targeting.* (Owner of a BlueStar clinic, Volta, Ghana)

However, it was unclear in both countries whether this increase ultimately translated into more profit, making the private facilities more financially viable and sustainable. Although we did not collect data that would allow us to corroborate these reports, preliminary results from a study among Amua providers in Kenya suggest that NHIF accreditation often does boost profits, although there is some differentiation according to NHIF contract type [57]. Similar data is not available for Ghana, though Ghanaian providers likely have more difficulty realizing profits under the NHIS due to payment delays and low reimbursement rates.

#### Patients: Perceived benefits of enrollment

While patients in both countries felt that having NHI coverage made healthcare more accessible, several patients in Ghana particularly appreciated having access to a wider variety of both providers and services through the NHIS. Patients in both countries overwhelmingly cited the caring, respectful treatment they received at private clinics and shorter wait times as the reasons why they would visit a private clinic over a public facility. In comparison to private providers, many clients thought that staff at public health facilities were at best over-worked and disinterested in serving patients, and at worst disrespectful.

*Those for the government, mostly if we go you get that you are there and the Doctor is busy concerned with other things, he is not even in a hurry with you. And like here when you just arrive here [the private clinic], you are attended to, mostly those doctors who are here are so many it’s not only one Doctor. Which problem do you have…* ***Like everyone wants to help you, they have the heart to assist you. So, we see that here is better than public*** *. (Patient at a Tunza clinic, Nyanza, Kenya)*

Indeed, one patient in Ghana said she valued the NHIS because it makes private healthcare more affordable, covering all of her services regardless of whether she is attending a public or a private facility. As one NHIF official in Kenya said, enrollees are “spoiled for choice” when more facilities become accredited and the competition this creates among providers can result in improved quality of care at clinics who are vying for clients.

Although patients in both Ghana and Kenya expressed an overall preference for private providers, clients in both countries perceived that they could access a wider range of services at a public hospital than they could at a private clinic, and often thought that staff at public facilities were better qualified than private practitioners. As a result, clients sometimes reported seeking out public facilities when they believed they were in need of more services or specialized equipment.

*Because there are some of the private hospitals, maybe the illness you are suffering from – let’s take for example, the big, big sickness around – when you go to the hospital that the company has recommend to you, they might not be having those particular machines there to use. But when you have nationwide, uh, National Health Insurance and go the government hospitals, they will serve you.* (Patient at a BlueStar clinic, Ashanti, Ghana)

Clients and community focus group participants in both countries also noted that public facilities are cheaper than private clinics. When weighed against the benefits of a private facility, though, cost was less of a concern than reliability and quality of treatment.

However, while patients in Ghana appreciated having more provider choice under NHIS and Kenyan patients expressed a preference for private providers, having NHIF coverage had much less effect on provider choice in Kenya than in Ghana. Kenyan patients were far less likely to consider whether or not they could pay with NHIF when selecting a clinic. Thus, patients enrolled in NHIF may not be realizing the benefit of greater provider choice to the same extent as their counterparts in Ghana.

### Challenges with the SHIs

#### Providers: Challenges under accreditation

Particularly in Ghana, providers often found that the anticipated financial benefits of accepting SHI coverage did not match their experience once accredited. While some Kenyan providers complained of delayed payments from NHIF, this problem was far more pronounced in Ghana. Providers interviewed there frequently cited payment delays as their greatest challenge with NHIS; a finding that held true across all three rounds of data collection. These delays ranged from 3-to-4 month delays in 2013 to providers in 2017 commonly reporting waiting 9-to-12 months for payment. In the face of these delays, providers often faced challenges stocking drugs and paying staff.

While the NHIA officials interviewed did not speak directly to these delays, they did confirm that the NHIA was facing financial difficulties. One official acknowledged an institutional problem with “sustainability,” stating that the organization’s funding had not increased in the 13 years since it was founded, although membership had grown over tenfold during this time.

In addition to payment delays, providers in both countries also complained that reimbursement rates were inadequate. In Ghana, providers complained that NHIS reimbursement rates were too low and needed updating to bring them in line with current market prices. Further, smaller providers faced challenges with the drugs and services reimbursed by NHIS. As noted above, these reimbursements are restricted according to provider type. Providers complained that such restrictions put smaller clinics at a disadvantage because they are only authorized to receive reimbursement for a smaller selection of drugs and services than they often provide. One NHIA official noted that inadequate funding was a common complaint, but that fraud was also a major concern from the NHIA side. This official felt that providers sometimes tried to submit claims multiple times in order to get more money, or that they did not understand why certain claims were rejected and felt they were being cheated by the NHIA as a result.

*So while as you were crying that our money is not enough, obviously in the insurance industry we still have people who want to abuse the system…So, NHIA over the years we’ve used what we call telecall audit system where we audit claims that are submitted to us…[In some cases], you were paid 10 million [Ghana Cedis] but we notice that 2 million of them were back claims. So, we deduct it from the claim that you submit.* (NHIA Official, Ghana)

#### Challenges with capitation schemes

In Kenya, outpatient NHIF coverage functions on a capitation system; a system that pools financial risk across an entire patient population and pays providers a regular lump sum based on the number of patients registered to their particular clinic, regardless of whether or not all patients require services during the defined period. In Ghana, the NHIS started a capitation pilot in the Ashanti region in 2012 and has plans to continue scaling up to other regions following review of the initial pilot, although these plans are currently on hold. We found that capitation posed some unique challenges for providers. Providers in both countries had difficulty understanding the guiding principles behind capitation, which led to misunderstanding of the system’s mechanics. Namely, rather than understanding capitation as a risk pool that covers an entire group of patients, providers instead saw the regular lump sum payments as a cap on the amount they were able to spend on an individual patient during the payment period.

*Interviewer: How much do they allocate under capitation for one patient maybe…?*

*Respondent: 500 [KSH], inclusive of lab.*

*Interviewer: One treatment or…?*

*Respondent: Yeah, one treatment. Not more than five hundred for outpatient.*

(Nurse/Midwife at a Tunza clinic, Eastern, Kenya)

However, while providers had difficulty understanding the financial aspects of capitation, they all understood that SHI payments are tied to the number of clients registered with their particular facility. As a result, a lack of understanding around facility registration among patients proved challenging at the clinic level. Several providers complained that patients came to their clinic expecting free treatment even though they were registered elsewhere. Further, some providers reported that they were losing clients to other facilities as a result of competition under capitation.

*The capitation is decreasing our savings…the other facility has taken all our customers.* (Midwife at a BlueStar clinic, Ashanti, Ghana)

In some cases, providers suggested that patients were registering with competing clinics that offered more services or had more highly qualified staff. There also was suspicion that larger clinics with more resources were recruiting clients and even paying their registration fees.

*The capitation is collapsing our work. It’s collapsing our work. And so we cannot get money to go and register for the clients to choose my facility. That’s what most doctors are doing. They go and pay for the cost of registration for the clients for their facility. Mhmm. And for us, the midwives, we do not have the money to be able to do that thing [pay for clients to enroll in NHIS].* (Owner at a BlueStar clinic, Ashanti, Ghana)

Conversely, NHI officials thought that capitation was good for providers, particularly those in the private sector. Recognizing that private providers have to cover all of their own costs and sometimes struggle to do this, officials from both NHIs noted that the regular payments with, in the case of Kenya, rates higher than those set for public facilities, should be a good thing for these providers.

*Private providers in terms of the capitation program supporting fully…to them it’s cash up front and they don’t get any support from the government in terms of payment of salaries for the staff. So that is the difference between them and the public providers; public provider’s staff get paid through government fund. So, it’s in the interest of the private providers to really get involved and support the capitation program, since on a monthly basis it becomes cash up front for them.* (NHIA Official, Ghana)

#### Effects of provider challenges with the NHIs

As a result of the financial challenges that providers faced participating in the SHI schemes, costs often were passed on to patients to cover out of pocket or services were limited, regardless of whether or not the services they were using were fully covered by insurance. In order to manage financial shortfalls caused by significant payment delays in Ghana, providers reported charging clients on top of NHIS reimbursement rates, operating on credit (particularly with pharmacies in order to stock drugs), or paying some clinic costs out of their own pocket. One provider had her own internal system that functioned like health insurance risk pooling, whereby she charged clients with minor ailments in order to cover the costs of more expensive treatments for others:

***The clinic must take money from one patient and use it to buy drugs to cater for another patient whose sickness is severe and at the verge of death****. Ahaaa. It may even happen that at the time, the patient at the verge of death may have no money, and must you leave that person to die? No. you will not leave that person. You must find something to do so that that person can also come back to life.* (Midwife at a BlueStar clinic, Ashanti, Ghana)

Some providers also mentioned that clients expect all of their services to be free under NHIS, but due to reimbursement restrictions for smaller providers, they cannot offer NHIS patients the same services they would receive at a larger facility. Patients therefore demand services for free that smaller providers cannot offer and some providers said they had lost clients as a result. Some providers also felt that these differential reimbursements forced them to treat patients differently according to their NHIS status, offering more comprehensive services to clients not covered by insurance.

Under capitation in Kenya, provider misunderstanding of the system often resulted in patients being charged when they required services or drugs that exceeded the monthly amount providers believed they were allotted. In instances where patients were not asked to pay, Kenyan providers regularly mentioned limiting the services they provided to clients paying with NHIF instead, so as not to risk losing money for their clinic.

However, while the accreditation process in both countries provides quality checks on providers by ensuring they have proper licensure, equipment, and resource capacity, none of the providers interviewed mentioned interacting with SHI officials after the accreditation phase unless they were applying for a renewal. Beyond processing claims, which is conducted in regional, county, or district centers, on-site monitoring from both the NHIF and NHIA appears to be minimal. Without regular oversight, providers may have more opportunities to levy charges at will.

#### Patient challenges with SHI coverage

The greatest challenge patients in both Ghana and Kenya faced under the SHIs was a lack of knowledge. This lack of knowledge resulted in patients being unsure if they were charged correctly at the clinic and affected their perceptions of the care they received. Indeed, knowledge of SHI coverage among clients interviewed in both Kenya and Ghana tended to be experiential; when asked which services were covered by the scheme they often cited services they had received themselves, but had little knowledge of specific services or programs beyond their own experience. Patients generally understood that NHI doesn’t cover all services or drugs, but often weren’t able to cite specific services that are or are not covered. Further, some clients were aware of their lack of knowledge regarding NHIS services and expressed a desire to learn more about NHIS coverage from their providers:

***We will also beg of them to tell us exactly what the health insurance covers****. Because sometimes when you go to the hospital you will be told that the health insurance does not cover “Drip” and some of the drugs too are not covered by it…it does not cover admission beds too. So, we don’t know what exactly the health insurance covers…* (Patient at a non-franchised clinic, Greater Accra, Ghana)

One NHIF official acknowledged the dearth of information passed on to enrollees, saying, *We do a lot, but I think our communication is not as good as it should be. We don’t say as much as we should*.

Corroborating provider reports of charging NHI-enrolled clients for drugs or services that should have been covered, patients regularly reported making some kind of out-of-pocket payment when they visited a clinic. This was particularly true in Ghana, where providers faced significant reimbursement delays. However, while patients often reported having paid for some services or drugs out of pocket when visiting the private clinic, they weren’t sure if they had been charged correctly. Among clients in Kenya, who knew less about NHIF coverage and did not expect completely free services under NHIF, satisfaction was higher with the amount they had paid in the clinic than their counterparts in Ghana. However, Kenyan patients also were more likely to report that they had paid nothing for their clinic visit, including drugs. While most patients in Ghana reported that the fees they paid felt reasonable, these patients were more likely to expect free services. This may be a result of having received more comprehensive coverage when the NHIS first started operating, and providers were paid adequately and on time.

Further, across rounds of data collection, patients in Ghana specifically expressed concern that NHIS doesn’t cover enough services or drugs, resulting in patients having to make additional cash payments at the clinic or visit a pharmacy to pay for medications out of pocket. A few patients reported that they didn’t even bring their NHIS card with them to the clinic because they felt it didn’t provide adequate coverage:

*Respondent: Mm, me, to me, right now I don’t have health insurance. I have the card alright but it’s at home. When I visit the hospital, I go with money.*

*Interviewer*: *Okay, okay, okay, why?*

*Respondent*: *Because when I even possess the health insurance it covers nothing. Mm.* (Patient at a Bluestar clinic, Ashanti, Ghana)

In this context, patients sometimes felt cheated by a system that they believed no longer functioned properly.

As a result, after initially enrolling in the SHIs, a number of patients in both countries reported allowing their enrollment to lapse when it came time to renew. In Ghana, for example, some patients decided not to re-enroll in the NHIS because they still paid at the clinic and therefore didn’t see any benefit in NHIS membership:

*So, after the treatment, the drugs prescribe – sometimes the doctor writes prescription and if you have [National] Health Insurance they will tell you they don’t have but when you are buying with cash they will be able to get some for you…So, I normally see it as, I registered for when the Health Insurance was introduced about 12 years ago, but as it expired I’ve not re-apply for it again.* (Patient at a Bluestar clinic, Ashanti, Ghana)

Among those who maintained enrollment, while a number of Ghanaian clients reported that they use NHIS to pay for services every time they go to the clinic, clients in Kenya did not report using NHIF every time they accessed health services. Ironically, and in contrast to complaints that NHIF fees were too expensive, some patients had only used NHIF one or two times even though they had been enrolled, and presumably paying the monthly fees, for years. Since NHIF fees are automatically deducted from workers’ salaries, it is possible that these interviewees did not have control over whether or not they paid for NHIF coverage, which would help to explain their continued payments. However, several patients apparently did not know how to use NHIF once they had it. These patients thought they had to bring their NHIF card with them to the clinic in order to receive services, although this is not the case, and so did not try to pay with NHIF when they didn’t have the card with them.

*Interviewer: Have you ever used NHIF since you got it?*

*Respondent: I have never.*

*Interviewer: Why?*

*Respondent:…I always assume that they want the card. When I went [to the NHIF office] last year I was told that the machine was broken…The machine was broken. The one that generate the card.* (Patient at a Tunza clinic, Eastern, Kenya)

Under Kenya’s capitation system, patients who paid for NHIF without using it may also have been registered with a particular clinic, but elected to receive services elsewhere; a hypothesis supported by our finding that NHIF-enrolled patients in Kenya were far less likely to seek out an accredited facility than their counterparts in Ghana.

Finally, SHI-enrolled patients in both countries disagreed on whether they were treated better or worse than cash-paying clients. In some cases, clients felt they were treated better or faster than patients paying with cash, which one Kenyan client suggested was because providers know that patients covered by NHIF are definitely able to pay. Conversely, some patients in both countries suggested that clients paying with cash received better services and higher-quality drugs than those paying with NHI.

Unlike providers, a number of whom were frustrated with the capitation pilot program in Ghana, or confused by the concept in Kenya, patients did not have strong reactions to the concept of capitation and were generally informed about the concept and the need to register with a particular clinic. However, patients had mixed reactions to the capitation model in terms of provider choice; some felt it gave patients the opportunity to choose the clinic where they wanted to seek treatment, while others suggested that capitation limits the ability to shop around.

## Discussion

Since Social Health Insurance schemes require private providers to achieve minimum standards of quality in order to become accredited and to maintain these standards through regular re-accreditation, these schemes help to ensure quality across the uneven terrain of private sector healthcare in Kenya and Ghana. However, as demonstrated above, ensuring access to these quality private providers through SHI coverage, particularly for low-income populations, is much harder. Issues such as lack of funds, provider and patient lack of understanding of the system, restrictions on reimbursements to small private providers, and inadequate infrastructure affect both cost of care and patients’ abilities to secure financial support for healthcare.

The World Health Organization points to the importance of taking a systems-level approach when attempting to serve a large population and particularly to address health inequities [58]. This approach provides a useful framework for addressing the challenges identified in Kenya and in Ghana. Indeed, while providers in Ghana faced a lack of funding from the NHIS and passed these costs on to patients as a result, simply funneling more money into the NHIS over the short term is not an adequate solution. As Duran et al. [59] point out in their analysis of India’s push toward UHC, money is necessary, but not sufficient for health system improvement. This is particularly true when there is no sustainable source of funding, resources are not allocated correctly, or there are no accountability mechanisms in place. Indeed, one official from the NHIA specifically noted in his interview that the NHIS faces a problem of financial “sustainability” given its long history and stagnant funding base.

Reich et al. [60] have argued that in order to achieve UHC, finances must not only be adequate, but must be managed correctly with attention paid to creating efficiencies in the system; an argument often used to support capitation [61]. However, building a health system that is responsive to conditions on the ground and that translates evidence to policy is just as important [59]. The data outlined above suggest that capitation may not be the best strategy if both the NHIA and NHIF hope to reach low-income patients with affordable healthcare. While this system may be easier to manage on the government end and should generate higher quality healthcare through increased competition, in reality we found that small private providers struggled to understand and implement a system of financial risk pooling within their own facilities, often charging patients on top of their SHI coverage as a result. However, patients were rarely aware of which charges were appropriate and which were not, and SHI officials did not appear to monitor these practices.

These findings align with other findings that capitation works best in a health system with a well-functioning and agile bureaucracy that can effectively monitor program implementation [62, 63]. Other forms of purchasing, such as the Diagnosis Related Group (DRG)-based payment system currently found in Ghana, may be a more effective way for government to purchase health services from private providers in such a scenario. A DRG-based payment system creates more efficiencies than a fee-for-service payment option while also allowing risk pooling at the national level, where it can best be managed [64, 65].

While the effects of capitation on the overall health markets in Kenya and Ghana (if fully implemented) will take a long time to play out, providers charging their patients informal fees on top of SHI coverage is a problem across both countries that is not limited to capitation facilities. These informal fees have significant potential to limit access to quality affordable healthcare for people living in poverty. Targeting informal fees through formal accountability mechanisms is therefore another approach SHI officials can take to increase access to a wider variety of providers. The literature on provider accountability for quality of care in LMICs suggests this can be a challenging endeavor [66], which can sometimes be mitigated by community involvement [67, 68] or by provider performance reports [69]. While these approaches have had limited success, Berlan and Shiffman [70] suggest they are promising.

In this case, the SHIs themselves offer a mechanism for this accountability. Indeed, the very purpose of strategic purchasing for health is to give the purchaser (the SHIs) power to elicit quality through payment. In the context of strategic purchasing, the literature suggests this is best done by setting clear guidelines, and putting strong systems in place to monitor provider performance and curb corruption [71, 72]. Since providers in both Ghana and Kenya value their accreditation status, our data suggests that providers would be motivated to charge correctly if their relationship with the SHIs was partially dependent on this performance measure. However, particularly in Ghana, these checks must be accompanied by increased funding and financial reform within the SHIs so that small private providers can be adequately funded through government financing.

## Conclusion

Our data point to several major barriers to SHI access and effectiveness for low-income populations in Ghana and Kenya, which in turn translated to challenges for patients trying to access quality health services from accredited providers. In addition, the data also pointed to opportunities to better engage private providers to serve low-income populations. On the one hand, patients in both countries knew that SHI enrollment could make healthcare more accessible by reducing cost and this incentivized many to register. However, it was not uncommon for patients to then allow their enrollment to lapse due to difficulty paying monthly fees (Kenya), or because they felt they didn’t realize the intended financial benefits when they visited the clinic (Ghana). Many patients in Ghana also complained that registration was burdensome due to long wait times and limited connectivity; a systemic issue corroborated by NHIA official reports. For those who used their SHI cards when visiting the private clinics, patients in both countries were unclear as to what exactly their coverage included. This uncertainty left clients confused about when it was appropriate to pay for services, leaving room for providers to levy out-of-pocket charges at will.

Despite the perceived challenges of accepting SHI and lack of clarity around the financial benefit to clinics, providers in both countries nonetheless felt market pressure to accept SHI coverage, and SHI officials similarly noted from a higher-level perspective that becoming accredited is becoming increasingly critical to sustainability for private providers. In Kenya, where outpatient coverage under the NHIF was limited until recently, providers found the accreditation process particularly challenging. In Ghana, where most providers already were accredited, providers struggled to cover costs in the face of inadequate or delayed reimbursements from the NHIS and sometimes passed these costs on to patients covered by NHIS or limited the services they received. Providers in both countries faced challenges understanding capitation, which also resulted in providers charging patients unnecessarily and offering them reduced services. SHI officials in Kenya should therefore consider replacing the capitation payment model with the DRG payment model used in Ghana, while NHIA authorities in Ghana should not plan to expand capitation country-wide. Further, both countries should consider building more monitoring and accountability mechanisms into their SHI systems in order to reduce potential costs to low-income patients while also ensuring that health facilities maintain the level of quality required to receive government financing.

## Abbreviations

AHME: African Health Markets for Equity
ERC: Ghana Health Services Ethical Review Committee
FGD: Focus Group Discussion
IPA: Innovations for Poverty Action
KEMRI: Kenya Medical Research Institute
LMIC: Low- or Middle-Income Country
MSI: Marie Stopes International
NHI: National Health Insurance
NHIF: National Hospital Insurance Fund (Kenya)
NHIS: National Health Insurance Scheme (Ghana)
PSK: Population Services Kenya
RCT: Randomized Controlled Trial
SHI: Social Health Insurance
UHC: Universal Health Coverage
